# Emerging Biomarkers in Urological Cancers: Angiogenesis and Damage-Associated Molecular Pattern Signaling

**DOI:** 10.3390/ijms26189130

**Published:** 2025-09-18

**Authors:** Kacper Robert Karpiuk, Grzegorz Młynarczyk, Joanna Matowicka-Karna, Barbara Darewicz

**Affiliations:** 1 Department of Urology, Medical University of Białystok, 15-276 Białystok, Poland; mlynarz36@yahoo.pl (G.M.); bdarewicz@op.pl (B.D.); 2 Department of Clinical Laboratory Diagnostics, Medical University of Bialystok, 15-269 Białystok, Poland; joanna.matowicka-karna@umb.edu.pl

**Keywords:** GDF15, VEGF, TGF-*β*1, HSP90, HMGB1, S100A9, DAMPs (damage-associated molecular patterns), angiogenesis, renal cell carcinoma, bladder cancer

## Abstract

The interaction between tumor cells and stroma in urological malignancies is governed by secreted and damage-associated factors that promote angiogenesis, immune modulation, and metastasis. This review synthesizes current evidence on six biomarkers—GDF15, VEGF, TGF-*β*1, HSP90, HMGB1, and S100A9—detailing their biological functions and clinical implications. We discuss GDF15’s roles in metabolic stress and immune regulation, VEGF’s central role in neovascularization, and TGF-*β*1’s dualistic tumor-suppressive and promotive effects. We then examine damage-associated molecular patterns, highlighting HSP90’s extracellular immunomodulation, HMGB1’s signaling via pattern-recognition receptors, and S100A9’s pro-inflammatory activity through RAGE and Toll-like receptors. Comparative analyses across renal cell carcinoma and bladder cancer cohorts elucidate each marker’s diagnostic accuracy, prognostic value, and predictive capacity for targeted therapies. Notably, GDF15 and HSP90 correlate with ferroptosis susceptibility in RCC and urinary VEGF with HMGB1 increases the chances of non-invasive bladder cancer detection. We suggest that multiplexed biomarker panels could enhance early detection, risk stratification, and personalized treatment in urological oncology. We advocate for prospective studies to validate thresholds, clarify interactions, and improve clinical integration.

## 1. Introduction

Cancer is a primary cause of morbidity and mortality globally, significantly influenced by the intricate interactions of genetic, environmental, and metabolic factors that collectively lead to the reprogramming of normal cells into a state of uncontrolled growth and spread [1]. The process is fundamentally reliant on the diverse array of bioactive molecules, including growth factors, cytokines, hormones, and damage-associated signals, that are secreted by both transformed cells and adjacent stromal components. The mediators coordinate essential characteristics of malignancy, such as continuous proliferative signaling, avoidance of immune detection, metabolic adaptation, and the initiation of neo-angiogenesis [2,3,4]. The increase in obesity and related metabolic disorders is associated with the dysregulated production of factors like growth differentiation factor 15 (GDF15), potentially connecting metabolic stress to oncogenic pathways [5]. These insights highlight the emergence of secreted and tissue-based biomarkers as valuable tools for improving early detection, risk stratification, and therapeutic monitoring.

Among urological malignancies, renal cell carcinoma (RCC) and bladder cancer each exemplify the urgent need for improved molecular diagnostics. RCC, typified by its clear-cell subtype, frequently exploits hypoxia-driven factors, such as vascular endothelial growth factor (VEGF), to secure an ample blood supply and support rapid tumor expansion [6,7]. Similarly, in bladder cancer, the high recurrence rates and heterogeneity of muscle-invasive vs. non-muscle-invasive disease have spurred investigations into stromal–epithelial interactions mediated by transforming growth factor beta 1 (TGF-*β*1) and other cytokines, aiming to predict progression more accurately than cystoscopy or cytology alone [8].

The release of damage-associated molecular patterns (DAMPs), including high-mobility group protein B1 (HMGB1) and members of the S100 family, has gained attention for their dual roles in creating an immunosuppressive microenvironment and activating innate sensors such as toll-like receptors (TLRs) and receptors for advanced glycation end products (RAGE) [9,10]. These signals not only perpetuate chronic inflammation but also support epithelial-to-mesenchymal transition, invasion, and pre-metastatic niche formation. Consequently, the integration of pro-angiogenic, pro-inflammatory, and stress-response biomarkers holds promise to generate a more nuanced picture of tumor biology, one that could guide personalized interventions and overcome the limitations of imaging and tissue biopsy alone.

In this review, we focus on describing the emerging evidence for GDF15, VEGF, TGF-*β*1, HSP90, HMGB1, and S100A9 as candidate biomarkers across renal and bladder cancers—highlighting their biological rationales, analytical performance characteristics, and prospective roles in clinical practice.

### Limitations

Other angiogenesis- and DAMP-related molecules (for example IL-33 and calreticulin), as well as immunoregulatory markers with recognized clinical relevance (e.g., PD-L1), were not reviewed in detail in the present manuscript. Their exclusion indicates the intentional limitations in scope and length of this focused review, rather than any deficiency in biological or clinical importance. We recognize these markers as significant candidates for future reviews and systematic investigations.

## 2. Background on Renal and Bladder Cancers

### 2.1. Renal Cancer

RCC represents a significant and growing public health challenge, accounting for approximately 3–5% of all adult malignancies [11]. It demonstrates a rising global incidence, ranking 14th among all cancer types with an estimated 434,840 new cases and 155,953 deaths in 2022, positioning it 16th in overall cancer mortality [12,13]. RCC exhibits significant geographic and demographic variability, with a higher prevalence in developed regions and a male-to-female ratio of approximately 2:1. The peak incidence occurs in individuals during their sixth to seventh decades of life [11]. The etiology of RCC includes both genetic and environmental factors: hereditary cases are linked to mutations in the von Hippel–Lindau (VHL) tumor suppressor gene, whereas modifiable risk factors such as cigarette smoking, obesity, and chronic hypertension significantly contribute to sporadic cases [14,15]. Increased exposure to trichloroethylene has also been implicated [16]. Histopathologically, clear cell RCC (ccRCC) predominates (approximately 90% of cases), although papillary, chromophobe, and collecting duct subtypes display distinct morphological and molecular features [17]. Standard diagnostic approaches rely on ultrasound, computed tomography, and magnetic resonance imaging—to detect renal masses, supplemented by percutaneous biopsy when histological confirmation is required. However, these methods may lack sensitivity for early-stage or metastatic disease [18,19]. Consequently, there is a rationale to expand the diagnostic methods to include circulating and tissue-based biomarkers—such as GDF-15, VEGF, TGF-*β*1, HSP90, HMGB1, and S100A9—that may improve early detection, risk stratification, and monitoring of therapeutic response. In the following sections, we will critically appraise the most recent evidence regarding these emerging biomarkers, their analytical performance, and their potential integration into clinical practice.

### 2.2. Bladder Cancer

According to the GLOBOCAN 2022 study, bladder cancer is ranked as the ninth most commonly diagnosed cancer, with 613,791 incidents and over 3 times the amount in men compared to women. Additionally, it ranks as the 13th leading cause of cancer-related mortality in both sexes [12]. The etiology is multifactorial, involving environmental exposures such as cigarette smoking, contact with aromatic amines, and arsenic-contaminated water, as well as genetic susceptibilities, including polymorphisms in genes related to carcinogen metabolism and DNA repair. Risk factors include chronic urinary tract infections, prolonged catheterization, and prior pelvic irradiation [20,21]. Histopathologically, the majority of cases are urothelial carcinomas exhibiting a spectrum from non-muscle-invasive lesions, such as papillary tumors and carcinoma in situ, to aggressive muscle-invasive subtypes characterized by frequent TP53 alterations. Standard diagnostic algorithms rely on white-light cystoscopy, urine cytology, and cross-sectional imaging modalities, which—despite high specificity—often lack sensitivity for early lesions and recurrence [22,23]. There is an increasing necessity to supplement traditional diagnostics with molecular and proteomic biomarkers to improve early detection and therapeutic stratification. In this context, emerging candidates such as GDF-15, VEGF, TGF-*β*1, HSP90, HMGB1, and S100A9 have garnered considerable attention for their roles in tumor progression, angiogenesis, immune modulation, and stress response. In the following sections, we will comprehensively examine the latest evidence on these biomarkers—including their biological rationales, analytical platforms, and potential clinical utility—thereby outlining a roadmap for their future bladder cancer management usefulness.

## 3. Cancer Microenvironment and Associated Molecular Factors

### 3.1. Tumor Microenvironment—Associated Factors with Pro-Angiogenic and Immunosuppressive Activity

The serum macrophage inhibitory cytokine–1 (MIC-1) or GDF15 is a product of cells which is a part of the transforming growth factor (TGF-*β*) superfamily. TGF-*β*1 is another essential component of this family, participating in numerous regulatory processes in cells and altered pathways, particularly in tumorigenesis [24]. Angiogenesis plays a significant role in the development of cancerous tissue, particularly through the action of VEGF. VEGF is known to induce vasodilation by upregulating nitric oxide synthase (NOS), resulting in increased nitric oxide production. Its primary functions include vasodilation, angiogenesis, and enhanced vascular permeability [25,26]. The mechanism of action is crucial in tumor growth, including glioblastoma multiforme, melanoma, breast, lung, head and neck, ovarian, gastrointestinal tract, and of course renal carcinomas [27,28,29].

Every of the previously mentioned factors plays a significant role in tumor development and growth. The GDF15 has been mentioned to play both anti-tumorigenic and protumorigenic role, but the VEGF has been identified as having a specific role in tumorigenesis. Nevertheless, the MIC-1 factor was pointed out to be at higher levels in breast cancer cells, pancreatic, gastric, and colorectal cancer cells [30,31,32,33,34].

**Figure 1:**
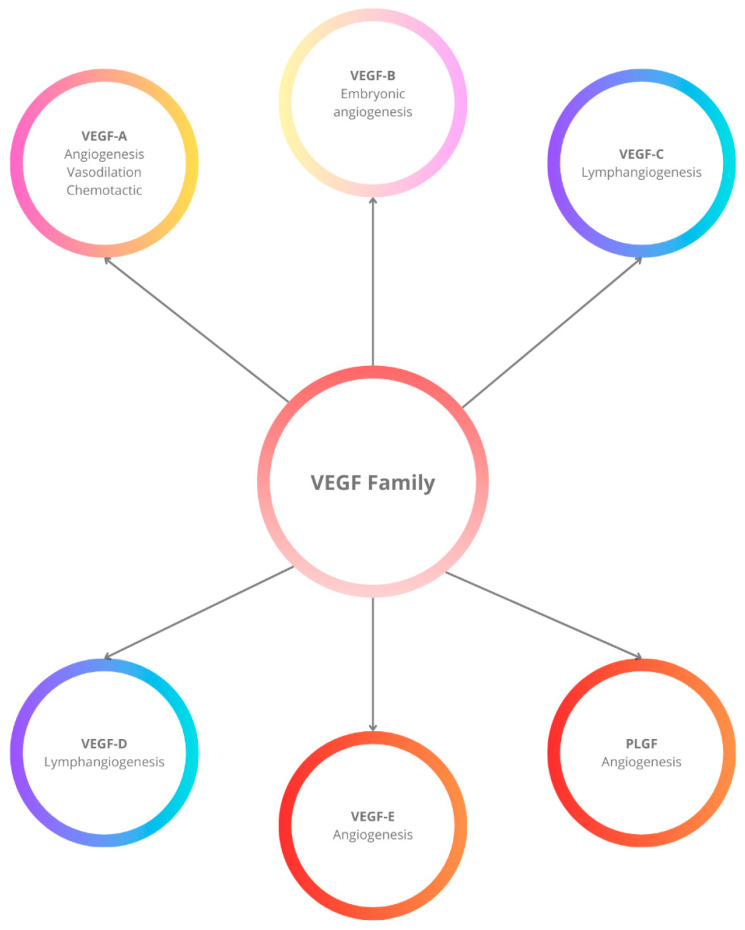
Members of the vascular endothelial growth factor (VEGF) family and their main biological functions. VEGF-A is primarily involved in angiogenesis, vasodilation, and chemotactic activity. VEGF-B plays a role in embryonic angiogenesis. VEGF-C and VEGF-D are key mediators of lymphangiogenesis. VEGF-E and placenta growth factor (PLGF) contribute to angiogenesis.

VEGF functions as a pro-angiogenic factor in the body, facilitating the formation of new blood vessels, which can contribute to cancer growth and expansion. It includes several isoforms: VEGF-A, VEGF-B, VEGF-C, VEGF-D, and VEGF-E, the latter of which is associated with parapoxvirus Orf and placenta growth factor [Figure 1]. VEGF-A is the most characterized member of the VEGF family [35]. The main stimulus for its expression is hypoxia, which build up the connection of hypoxia-inducible factor-1*α* (HIF-1*α*) to VEGF promoter, which then increase the expression of VEGF [36,37].

VEGF plays a crucial role in various types of cancer. For instance, one of them is breast cancer, where one of the studies showed that VEGF alongside other factors can induce cancer cells stemness [38]. A separate study indicated that autocrine secretion of VEGF-A mediated tumor cell growth in non-squamous cell lung carcinoma [39]. Considering the studies described, it can be concluded that VEGF may directly influence tumor cell growth, invasiveness, and survival.

There is also cytokine, that nature has not strictly assigned to tumor suppressive or tumor promoting mechanisms. TGF-*β* is a frequently expressed cytokine that acts in various cancer types. The TGF-*β* superfamily comprises a significant collection of proteins essential for various cellular functions, including bone morphogenetic protein, growth differentiation factors (GDFs), Mullerian inhibitory factor, glial-cell-line-derived neurotrophic factor, TGF-*β*1, TGF-*β*2, TGF-*β*3, activin beta chains, the protein nodal, and many more [24,40]. TGF-*β* work as an tumor suppressive agent especially in early stages of tumorigenesis, by modulating cell apoptosis and proliferation. One of the mechanisms that it is working in is the inhibition of cell cycle by arresting cell in G1-phase through activating cyclin dependent kinase inhibitors p15 and p21 or by suppressing c-Myc oncogene [41,42]. On the other hand, TGF-*β* can be a major factor in tumorigenesis, especially in later stages of the disease [42]. Many of the tumors are reported to take part in such a process, including breast, colon, and prostate cancer [43,44,45,46,47]. This activity mechanism involves the stimulation of epithelial-to-mesenchymal transition (EMT), along with cell invasion, metastasis, and proliferation [48]. One of the more important members of the TGF-*β* superfamily is TGF-*β*1. The receptor TGF-*β*R1 plays a crucial role in tumorigenesis, such as by upregulating matrix metalloproteinase 9 in human melanoma cells [49]. In conclusion, the TGF-*β* proteins are found in a plethora of tumors, so the further detailed study of their associations and actions in these diseases is essential to develop new therapeutic and diagnostic strategies.

### 3.2. Danger Signals: Molecular Patterns of Damage in Cancers

**Figure 2:**
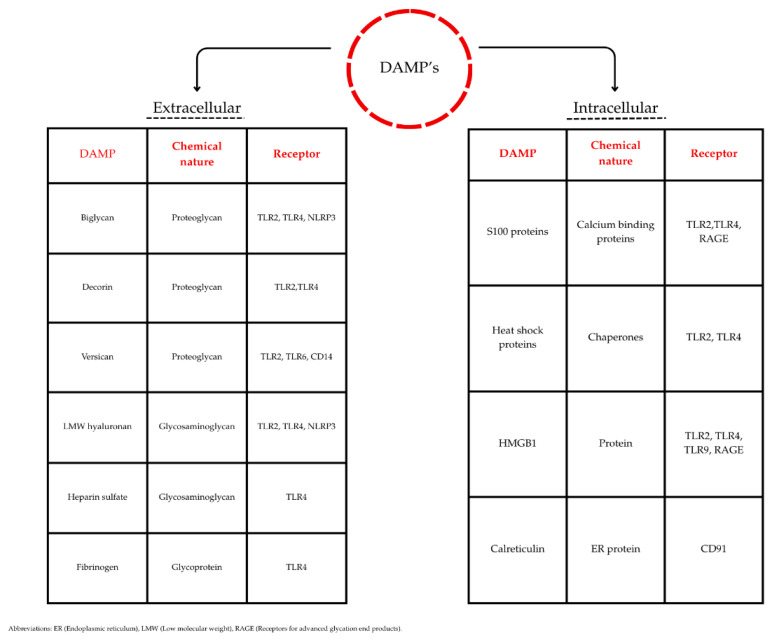
Classification of selected extracellular and intracellular DAMPs with their chemical nature and primary pattern recognition receptors (PRRs).

DAMPs are molecules released by necrotic or simply damaged cells, which then start the immunological response. By binding to the pattern recognition receptor (PRR), these molecules can stop further breakage by inhibiting damaging factors and initiating healing processes [50]. DAMPs originate either from extracellular or intracellular matrix throughout stress of the tissue and death or injury of the cells. By this division, we can separate two main groups: (1) DAMPs originating from the extracellular matrix, which are Biglycan, Decorin, Versican, low molecular weight hyaluronan, Tenascin C, Fibrinogen, and Heparan sulfate Fibronectin-EDA; (2) Intracellular DAMPs, which include Uric acid, S100 proteins, heat shock proteins (both of the previously mentioned proteins are originating from the cytosol), Syndecans, Glypicans, mtDNA, mROS, ATP, Formyl peptide, mitochondrial transcription factor A, Histones, DNA, IL-1*β*, and the notable factor HMGB1 [Figure 2]. DAMPs initiate the inflammatory and destructive processes via multiple receptors. These receptors can be found in many of the human tissues, because the inflammation process can begin almost anywhere. The receptors for DAMPs are mainly categorized as a group of TLRs and the RAGE [51,52].

DAMPs that have garnered increasing attention in the cancer research domain in recent years include the S100 protein family, the heat shock protein (HSP) family, and HMGB1 [53,54,55,56,57]. HMGB1 has been demonstrated to be released from necrotic cancer cells into the microenvironment. It regulates the processes of inflammation and corrective responses that, in the cancer habitat, can lead to metastases, cancer cell survival, and expansion [58]. Several studies showed a significant connection between HMGB1 activity and poor prognosis across various cancers, including breast, prostate, colon, and pancreas cancer [59,60,61,62,63]. In mouse models, genetic deletion or pharmacologic blockade of RAGE suppressed tumorigenesis in skin and liver cancer [64,65]. Also, HMGB1 is capable of promoting activation and releasing processes of intratumoral T cells, which in return recruit waves of cancer-inducing macrophages [66]. HMGB1 plays a crucial role in the process of tumorigenesis by promoting immunosuppressive and inflammatory pathways.

Another member of the DAMP family that is worth mentioning is the heat shock protein 90 (HSP90) group. The increased expression of HSP90 is initiated by the activation of corresponding genes in response to heat stress [67]. Members of the heat shock protein 90 family are various, ubiquitous molecules with approximate molecular weights of 90 kDa. Taking into account the newest nomenclature regarding the HSP, the HSP90 family consists of five members, collectively named HSPC [68]. Another classification of this group is by their cellular localization, including HSPC1 (HSP 90-*α*), HSPC2 (HSP 90-*α* A2), and HSPC3 (HSP 90-*β*), ER resident member-HSPC4, GRP94 (GP96) and HSPC5 (TRAP1) [69]. This review focuses on HSPC1-3 factors due to the ongoing research surrounding them. Proteins which are potential target for HSP90 activity have been reported to appear in almost every type of cancer. The survival and proliferation of cancer cells heavily rely on the function of HSP90 molecular chaperones, which maintain the structural integrity of key oncoproteins [70]. It was shown that the level of HSP90 greatly correlates with the risk of increased malignancy and poor responsive to treatment in breast cancer [71]. Moreover, higher expression levels of HSP90 correlated with survival outcome in this type of cancer and was determining factor in survival probabilities [72]. Elevated levels of HSP can also be observed in the cancer cells of non-small-cell lung carcinoma; however, further research still need to be carried out [73].

Among the DAMP list, we identified S100A9 protein as an additional subject requiring examination. This protein is part of the S100 leukocyte proteins family, which can be secreted by immune cells, such as monocytes, neutrophils, dendritic cells, and activated macrophages, especially during damaging processes and inflammation, whereas in healthy circumstances, the levels of such proteins are persisting at lower levels [74,75]. S100A9, similar to previously discussed DAMPs, can interact with TLRs and RAGE receptors. In murine models, S100A8/A9 were shown to act as endogenous TLR4 ligands, triggering strong inflammatory responses. Signaling and connecting with these receptors can lead to maintaining the process of inflammation and the development of tumor cell differentiation and proliferation [76,77]. The operational structure of S100A9 can be observed in numerous carcinomas. For example, S100A9 contribute to promoting tumor growth in hepatocellular carcinoma by phosphorylation of ERK1/2 and P38 via binding to RAGE receptor [78,79]. The S100A8/A9-RAGE-NF-κB/MAPK pathway is implicated in the cell invasion of pancreatic cancer [80]. Interestingly, the S100A8/A9¬–MCAM–ETV4–ZEB1 axis is promoting the aggressiveness of breast cancer [81]. Furthermore, recent studies suggest that S100A8 and S100A9 function as transcriptional coactivators in the regulation of gene expression during the transformation of breast cancer cells [82]. It is worth adding that the S100A9 is capable of binding to the TLR4 receptor and then activate the NF-*κ*B pathway, which in result promotes cells to secrete fibronectin. In the next step, the signaling of *β*1-FAK facilitates the invasion of prostate cancer cells [83]. In conclusion, S100A9 is capable of binding to certain receptors, including TLR4, RAGE, and MCAM, through which it can trigger specific pathways which can then stimulate cancer cell proliferation, metastases, and malignancy.

## 4. Molecular and Cellular Factors Shaping the Cancer Microenvironment in Kidney and Bladder Tumors

### 4.1. GDF15 in RCC

Few research papers, particularly original studies, address the relationship between GDF15 and RCC. In recent studies, it has been observed in animal models that GDF15 serum levels increase after cisplatin administration, a highly nephrotoxic drug, thus bringing up the supposition that this factor can play a significant role as a nephrotoxicity marker during cisplatin treatment [84]. On the other hand, in a follow-up animal study, administration of candesartan, an angiotensin II receptor blocker, further increased GDF15 levels. According to that, the inflammation processes which takes place during the tumor expansion can be weakened and the kidney injury may be improved [85]. Focusing on ccRCC, research by Yang et al. revealed that GDF15 expression is significantly lower than in other renal carcinomas. This downregulation directly hinders ferroptotic cell death, facilitating increased tumor cell survival and proliferation. Lower GDF15 levels are significantly associated with more aggressive disease characteristics and a worse overall prognosis, establishing GDF15 as an independent prognostic biomarker in ccRCC. Notably, restoration of GDF15 expression reactivates ferroptosis pathways in ccRCC cell lines, suggesting that therapeutic strategies aimed at upregulating GDF15 or mimicking its ferroptosis-inducing activity may offer novel treatment directions for patients with GDF15-deficient tumors [86].

### 4.2. VEGF in RCC

The treatment of RCC with anti-VEGF medicines, such as bevacizumab, a VEGF-A neutralizing antibody, has been known for years, due to their potential as therapeutic agents [87]. Expression of VEGF in the microenvironment of the tumor leads to more frequent occurrence of tumorigenesis and metastases [88]. In RCC, VEGF-A expression is markedly elevated compared with normal kidney tissue and correlates strongly with higher tumor stage and poorer patient survival. Bioinformatic network analysis revealed that VEGF-A and its co-expressed neighbors participate in key oncogenic processes—including protein methylation, glycosylation, and cell adhesion—and are regulated by transcription factors such as HIF1A, TFAP2A, and STAT3. Furthermore, VEGF-A levels positively associate with infiltration of CD8^+^ T cells, macrophages, and other immune cells within the tumor microenvironment, highlighting its dual function as a prognostic biomarker and potential therapeutic target in RCC [89]. The role that VEGF plays in the RCC development and genesis is one of the most important courses that future studies need to delve into.

### 4.3. TGF-β in RCC

TGF-*β* and its signaling pathways have been confirmed to be present in the RCC in multiple studies over the years [90,91,92,93]. The research conducted by Che et al. elucidates the connections between RCC and TGF-*β*. In ccRCC, an integrated genomic and transcriptomic analysis of 539 TCGA samples revealed widespread dysregulation of the TGF-*β* signaling pathway, with 76 out of 85 pathway genes significantly altered compared to normal kidney tissue. Of these, 55 genes were identified as either protective or risk factors influencing patient prognosis, underscoring the dualistic role of TGF-*β* signaling in ccRCC biology. Unsupervised clustering based on TGF-*β* gene expression stratified tumors into high- and low-score groups, with the low-score cohort exhibiting significantly shorter overall survival, demonstrating that reduced TGF-*β* activity is independently associated with poorer outcomes. Moreover, low TGF-*β* scores correlated with decreased expression of key tumor suppressors such as VHL, PTEN, and TP53, providing in-depth insight into the aggressive phenotype of these tumors. Importantly, differential drug sensitivity analyses indicated that TGF-*β* score stratification could guide targeted therapy selection, as high-score tumors exhibited distinct responses to approved agents compared to low-score tumors [94].

In another comprehensive study, Takahara et al. demonstrated that TGF-*β*1 mRNA expression is significantly elevated in ccRCC tissues compared to adjacent normal kidney tissues, with high TGF-*β*1 levels correlating strongly with higher WHO/ISUP nuclear grade, increased tumor necrosis, and an enhanced likelihood of distant metastasis. Patients with elevated TGF-*β*1 expression exhibited significantly shorter relapse-free survival, establishing TGF-*β*1 as an independent prognostic marker of poor outcome. Furthermore, high TGF-*β*1 levels were associated with an inflammatory tumor microenvironment characterized by increased infiltration of FOXP3-positive regulatory T cells, plasma cells, PD-L1-positive immune cells, and the formation of tertiary lymphoid structures. These immune features, particularly the presence of PD-L1-positive cells, are known to impact the responsiveness to immune checkpoint inhibitors. Although TGF-*β*1 expression was not directly linked to the classic tumor immune microenvironment classification (inflamed, excluded, desert types), its association with immunosuppressive markers suggests a significant role for TGF-*β*1 in facilitating immune evasion. Overall, the findings highlight TGF-*β*1 as not only a biomarker of tumor aggressiveness and metastatic potential in ccRCC but also as a promising therapeutic target, especially in strategies aiming to enhance the efficacy of immune checkpoint blockade therapies [95].

### 4.4. HSP90 in RCC

In the context of DAMPs, particularly HSP90 in ccRCC serves as a significant molecular chaperone and an immunologically active DAMP that affects tumor progression and the microenvironment. Comprehensive research analysis of TCGA data managed by Liao et al. revealed that among the HSP families, HSP90B1 is one of eight members significantly upregulated in tumor vs. normal kidney tissue. It is a key component of a six-gene prognostic signature (HSPA8, HSP90B1, HSPA7, HSPA12B, HSPA4L, HSPA1L) that stratifies patients into high- and low-risk groups with distinct overall survival outcomes. Elevated HSP90B1 expression is clinically associated with advanced tumor stages and reduced survival, highlighting its role in promoting aggressive ccRCC phenotypes. Functionally, HSP90 family members facilitate ccRCC cell proliferation, invasion, and metastatic potential, as confirmed by in vitro assays, while bioinformatic characterization of the “high-risk” subgroup—marked by HSP90B1 overexpression—demonstrates an immunosuppressive microenvironment: increased infiltration of regulatory T cells (Tregs), higher PD-L1-positive immune cell counts, and altered cytokine profiles that collectively suppress anti-tumor immunity. This dual role—chaperoning oncogenic client proteins (e.g., HIF-1*α*, NF-*κ*B) and acting extracellularly as a DAMP to modulate immune cell recruitment—positions HSP90 as a bridge between tumor-cell-intrinsic survival mechanisms and the host immune response. Taken together, these findings establish HSP90, and particularly HSP90B1, as a prognostic biomarker and a functional DAMP in ccRCC, whose targeting may not only disrupt tumor cell homeostasis but also reprogram the tumor immune microenvironment toward enhanced immunogenicity [96].

Inhibition of HSP90 can also be observed as major step in treatment of another renal tumor, specifically papillary renal cell carcinoma (PRCC). In the realm of kidney cancer, particularly PRCC, the multifaceted role of HSP90 has garnered significant attention. Research by Pahwa et al. demonstrated that HSP90 inhibition, using the specific inhibitor SNX2112, elicits potent anti-tumor effects in PRCC models, evidenced by the induction of apoptosis, G2/M cell cycle arrest, and suppression of oncogenic pathways such as PI3K/AKT and MEK/ERK1/2. Beyond its well-established intracellular chaperone functions, HSP90 is capable of stimulating immune responses when released extracellularly. Although the study primarily focuses on intracellular mechanisms, the established immunogenic properties of HSP90 suggest that its inhibition may also modulate the tumor microenvironment in PRCC, potentially enhancing anti-tumor immunity alongside direct tumor cell suppression. This dual functionality underscores HSP90’s therapeutic significance and highlights the need for further exploration into how its role as a DAMP could inform innovative immunotherapeutic strategies for PRCC and other renal malignancies [97].

### 4.5. HMGB1 in RCC

There is limited evidence available regarding the correlation between HMGB1 and RCC. A comprehensive search can yield valuable discoveries, such as those made by Solakhan et al. In this study, they demonstrated that urinary HMGB1 is markedly elevated in patients with RCC compared both to healthy controls and to subjects with benign urinary tract infections (median 147.15 vs. 24.8 and 39.45 pg/mL, respectively; *p* < 0.001). HMGB1 levels correlated strongly with tumor burden and aggressiveness: values rose in parallel with increasing tumor diameter (r = 0.874, *p* < 0.001) and were highest in sarcomatoid variants—whose median HMGB1 exceeded 499 pg/mL—underscoring its association with high-grade histology (*p* = 0.035 for subtype differences; *p* = 0.002 for diameter comparisons). Receiver-operating characteristic analysis demonstrated an AUC of 0.897 for distinguishing RCC from controls and 0.818 vs. urinary tract infections (UTI), supporting strong diagnostic discrimination. Interestingly, they proposed that necrotic or stressed tumor cells release HMGB1 into the urinary space, where it may engage pattern-recognition receptors (e.g., RAGE, TLR4) to sustain a pro-inflammatory, tumor-promoting environment. Still, the study included a small RCC cohort (≈34 patients) and short follow-up; handling and single-center case selection may bias cut-off estimates. External validation in larger, prospectively collected RCC cohorts is therefore required. Collectively, these data position urinary HMGB1 as a non-invasive biomarker with both diagnostic and prognostic utility in RCC, particularly for identifying high-grade, aggressive disease. Further validation in larger, longitudinal cohorts—and integration with serum and tissue HMGB1 measurements—will be essential to confirm its role in patient stratification and monitoring [98].

### 4.6. S100A9 in RCC

Few studies elucidate the relationship between the S100A9 protein and RCC. An observational study by Koh et al. involving a cohort of 152 patients (294 evaluable tumor cores) with ccRCC provides significant insights into this connection. The study found high S100A9 expression in 37 cores, which correlated strongly with advanced T stage (*p* < 0.001) and higher Fuhrman nuclear grade (*p* < 0.001). Multivariate Cox regression confirmed that elevated S100A9 is an independent predictor of poor outcomes, with hazard ratios of 2.42 (95% CI 1.04–5.62; *p* = 0.039) for disease-free survival and 2.43 (95% CI 1.13–5.21; *p* = 0.023) for disease-specific survival. Intriguingly, tumor-derived S100A9—released as a DAMP—likely interacts with RAGE and TLR4 on both cancer and stromal cells, triggering NF-*κ*B and MAPK/ERK signaling cascades that promote a pro-inflammatory microenvironment, enhance tumor cell proliferation and facilitate pre-metastatic niche formation. Koh et al. provide a reasonably large tissue-based series (*n* ≈ 152) showing prognostic associations for S100A9, but the retrospective, single-center design and reliance on immunohistochemistry scoring limit causal inference and introduce possible selection and scoring bias; independent validation (ideally with multi-center cohorts) would be advisable [99].

**Figure 3:**
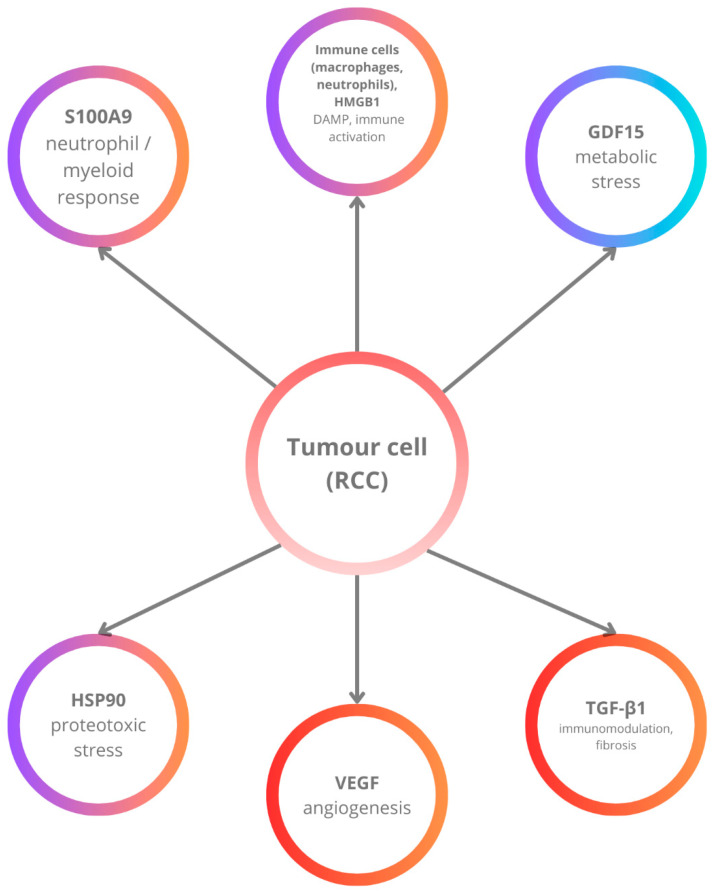
Simplified schematic overview of six emerging biomarkers in the RCC microenvironment. Tumor cells interact with angiogenic mediators (VEGF), immunomodulatory cytokines (TGF-*β*1), metabolic regulators (GDF15), and damage-associated molecular patterns (HSP90, HMGB1, S100A9). Collectively, these pathways promote angiogenesis, immunosuppression, metabolic adaptation, and inflammatory myeloid cell recruitment, driving RCC progression and therapy resistance.

The comprehensive research by Zhang et al. demonstrated that S100A9 is significantly overexpressed in the serum and tumor tissues of patients with early-stage (T1a) RCC when compared to benign renal lesions and healthy controls. Quantitative proteomics (iTRAQ) and mRNA analyses revealed that S100A9 levels rise in parallel with tumor stage, implicating its involvement in the transition from localized to more advanced disease. Immunohistochemical validation confirmed strong S100A9 staining within RCC cells, underscoring its potential utility as a non-invasive biomarker for early detection. S100A9’s capacity to interact with RAGE and TLR4 receptors on tumor and myeloid cells indicates its role in fostering a pro-inflammatory microenvironment. This interaction promotes leukocyte recruitment, MAPK activation, and the suppression of anti-tumor immunity, thereby facilitating the progression and metastasis of RCC. Taken together, these findings position S100A9 not only as a sensitive indicator of RCC presence and stage but also as a major link between DAMP-mediated inflammation and renal tumor biology, making it a promising target for diagnostic and therapeutic strategies in RCC [100] [Figure 3].

### 4.7. GDF15 in Bladder Cancer

Bladder cancer remains one of the most common cancer types among the world population, both men and women combined [101]. Nevertheless, recent studies examining the correlation between GDF15 and bladder tumors are limited, indicating the need for further research in this area, as demonstrated by the work of Chang et al. This study elucidates the important role of GDF15 in modulating the tumor microenvironment of bladder cancer. GDF15 expression is notably downregulated under hyperglycemic conditions, which are characteristic of diabetes mellitus, suggesting a link between metabolic dysregulation and bladder cancer progression. Conversely, pharmacological agents such as metformin and caffeic acid phenethyl ester can induce GDF15 expression through activation of the AMP-activated protein kinase pathway. This induction leads to the suppression of EMT markers and attenuates the proliferation and invasion of bladder cancer cells. These findings underscore the therapeutic potential of targeting GDF15 pathways to modulate the tumor microenvironment and inhibit bladder cancer progression. Importantly, the study also highlights that the loss of GDF15 under diabetic conditions contributes to a permissive environment for tumor expansion by enhancing stromal cell migration, which may further facilitate cancer dissemination. The reestablishment of GDF15 expression reverses stromal activation and restores epithelial characteristics in cancer cells, countering EMT-associated phenotypic changes. In this context, GDF15 functions as a multifaceted regulator influencing metabolism, inflammation, and tumor biology. GDF15, due to its sensitivity to metabolic signals and its function in inhibiting tumor-promoting stromal dynamics, presents a valuable molecular target for comprehensive therapeutic approaches, especially in individuals with metabolic comorbidities like diabetes [102].

### 4.8. VEGF in Bladder Cancer

In addition to GDF15, factors such as VEGF have been associated with bladder cancer. Notably, in recent years, an interesting study by Ayati et al. investigated this connection by assessing VEGF levels in serum and urine from 46 bladder cancer patients and 38 healthy controls. Their findings revealed no significant difference in serum VEGF concentrations across control (478.7 pg/mL), low-grade (518.4 pg/mL), and high-grade (648.1 pg/mL) groups (*p* = 0.175), suggesting that systemic VEGF may not reliably reflect tumor activity. Conversely, urinary VEGF levels were significantly elevated in bladder cancer patients—968.3 pg/mL in low-grade and 848.4 pg/mL in high-grade cases compared to 414.2 pg/mL in controls (*p* = 0.010)—indicating a localized release mechanism within the bladder microenvironment. This disparity underscores the hypothesis that VEGF may be preferentially excreted or concentrated in urine, potentially signaling angiogenic and inflammatory processes inherent to the tumor site. The increased levels of urinary VEGF in low-grade tumors indicate its potential as an early diagnostic biomarker, providing a non-invasive alternative to cystoscopy, which has variable sensitivity. Moreover, VEGF may indicate interactions with immune cells, such as macrophages or neutrophils, within the tumor microenvironment, potentially intensifying inflammation that supports cancer progression. This study also showed that specimen type and local tumor shedding significantly affect VEGF measurements. Additionally, studies linking VEGF to imaging-based staging (VI-RADS) suggest that VEGF levels track with invasion depth, so VEGF performance must be interpreted in the context of tumor stage/subtype and the chosen biofluid. The authors detected higher urinary VEGF in bladder cancer but the study was a relatively small, single-center case–control series (46 cases); the authors caution that sample size and single-center recruitment may explain inconsistencies with other reports. While the authors found no correlation between urinary VEGF and tumor grade or recurrence, the significant elevation in bladder cancer patients aligns with broader evidence linking VEGF to poor prognosis across various malignancies. These findings advocate for further studies to measure how VEGF influences bladder cancer’s immune landscape and to validate its clinical potential as a biomarker or therapeutic target, possibly through anti-VEGF agents that disrupt both angiogenesis and immune signaling pathways [103,104].

A detailed observational study was conducted by Bardowska et al., which provided evidence that urinary VEGF, among other biomarkers, plays a potentially significant role in stratifying the risk of recurrence and progression in patients with non-muscle invasive bladder cancer (NMIBC) following transurethral resection of bladder tumor (TURBT). Urinary VEGF concentrations were significantly higher in patients who experienced disease recurrence or progression compared to those who remained tumor-free, with a *p*-value of 0.0011. Although VEGF alone demonstrated only modest predictive accuracy (AUC = 0.632), its performance improved markedly when integrated into multivariable logistic regression and machine learning models, achieving an AUC of up to 0.84. Although prospective, Bardowska et al.’s cohort analysis found limited standalone prognostic value for single biomarkers (including urinary VEGF); the study highlights that modest single-marker performance and the need for multivariable models. Moreover, the current analysis requires external validation before clinical application. However, this study suggests that while VEGF may not independently serve as a standalone prognostic marker, it contributes valuable biological information when considered alongside clinical variables and other biomarkers [105].

There was also interesting research provided by Ponukalin et al., who observed simultaneous assessment of Vesical Imaging-Reporting and Data System (VI-RADS) and several growth factors and mediators of cell growth, including VEGF and TGF-*β*1. Their study provided evidence that both TGF-*β*1 and VEGF are significantly upregulated in patients with muscle-invasive bladder cancer (MIBC), supporting their role as critical mediators of tumor progression within the evolving bladder cancer microenvironment. Serum concentrations of both factors were found to rise in parallel with tumor invasion depth and local advancement, as assessed by multiparametric MRI and VI-RADS scoring. Specifically, patients with locally advanced and metastatic tumors exhibited a 2.2- to 2.9-fold increase in TGF-*β*1 and VEGF levels compared to healthy individuals, highlighting their value as circulating indicators of aggressive disease. TGF-*β*1 appears to facilitate immune escape by promoting a shift away from pro-inflammatory cytokine profiles and favoring the recruitment of immunosuppressive cell populations. Simultaneously, VEGF not only drives neovascularization but also contributes to immunosuppression by impairing dendritic cell maturation and fostering a hypoxic microenvironment conducive to tumor cell survival. The concurrent increase of TGF- *β*1 and VEGF indicates a remodeling of the tumor microenvironment, characterized by heightened epithelial–mesenchymal transition (EMT), immune evasion, and increased metastatic potential. Importantly, the study demonstrated that the combined diagnostic performance of TGF-*β*1 and VEGF was superior to that of either biomarker alone, achieving high sensitivity and specificity in distinguishing MIBC from non-muscle-invasive disease. These findings suggest that measuring serum levels of TGF-*β*1 and VEGF may provide a non-invasive means of identifying patients with high-risk bladder cancer and offer insight into the tumor’s inflammatory and angiogenic state [104].

### 4.9. TGF-β in Bladder Cancer

A comprehensive search revealed research by Efiloğlu et al., which elucidated the significance of TGF-*β*1 in NMIBC, underscoring its potential as a biomarker for disease progression. The findings indicate that lower serum TGF-*β*1 levels are significantly associated with an increased risk of NMIBC progression, suggesting a critical role for TGF-*β*1 in modulating tumor behavior. Specifically, the observed correlation between reduced TGF-*β*1 levels and progression underscores its tumor-suppressive function in early-stage NMIBC, potentially through mechanisms such as regulation of cell proliferation, apoptosis, and epithelial–mesenchymal transition. However, the lack of association between TGF-*β*1 levels and disease recurrence suggests that its utility may be more specific to progression rather than recurrence in NMIBC. Additionally, the study’s findings on the interplay between TGF-*β*1 and systemic inflammatory markers, such as the neutrophil-to-lymphocyte ratio, further emphasize the complex microenvironmental interactions influencing bladder cancer outcomes. These results advocate for the inclusion of TGF-*β*1 assessment in risk stratification models for NMIBC, potentially enhancing personalized treatment strategies [106].

Another detailed study was performed by Stojnev et al. significantly advances our understanding of the role of TGF-*β*1 in urothelial bladder cancer (UBC), highlighting its prognostic importance in a cohort of 404 patients followed for a median of 61 months. Through immunohistochemical analysis, the study found that high TGF-*β*1 expression, observed in 68.1% of tumors, is strongly associated with aggressive disease characteristics, including high-grade tumors (70.5%) and advanced stages (77.7% in pT2 muscle-invasive tumors compared to 45.4% in pTa non-invasive tumors). This suggests that TGF-*β*1 plays a critical role in promoting tumor progression, likely through EMT, which facilitates invasion and metastasis in advanced UBC. Notably, high TGF-*β*1 expression was an independent predictor of worse overall survival (hazard ratio 1.565, *p* = 0.041) and correlated with cancer-specific death (37.1% in TGF-*β*1-high patients, *p* = 0.043), underscoring its adverse prognostic impact. However, TGF-*β*1 did not significantly influence recurrence-free survival (*p* = 0.681), indicating its primary relevance to disease progression and mortality rather than tumor relapse. The study also revealed a gender disparity, with higher TGF-*β*1 expression in female patients (76.1% vs. 65.7% in males, *p* = 0.038), suggesting potential sex-specific differences in UBC biology. TGF-*β*1 may have tumor-suppressive effects in early, non-invasive tumors, contrasting with its oncogenic role in advanced stages, indicating its context-dependent functionality. These findings advocate for the integration of TGF-*β*1 expression assessment into clinical practice to enhance risk stratification and guide treatment decisions, particularly for patients with high-grade or invasive UBC who may benefit from aggressive therapies like chemo/radiotherapy (22.5% in TGF-*β*1-high patients, *p* = 0.017). Future research should focus on validating these results in diverse cohorts, elucidating TGF-*β*1’s immunomodulatory effects in the tumor microenvironment, and exploring targeted therapies against TGF-*β* signaling to improve outcomes in advanced bladder cancer. This study underscores TGF-*β*1 as a key biomarker with significant implications for personalized UBC management. TGF-*β*1 may be tumor-suppressive in early NMIBC but tumor-promotive in advanced disease. As Stojnev et al. and Efiloğlu et al. demonstrated, TGF-*β*1 associations differ by stage and clinical endpoint (progression vs. recurrence), so biomarker utility requires stratified analysis by stage/grade and careful endpoint selection [106,107].

### 4.10. HSP90 in Bladder Cancer

Research on certain DAMP’s has introduced many new developments regarding the relationship between HSP90 and bladder cancer. The research by Song et al. offered a comprehensive analysis of immunogenic cell death (ICD) in bladder cancer, highlighting the crucial function of HSP90AA1 in influencing the tumor immune microenvironment. By classifying 403 bladder cancer patients from the TCGA database into two ICD-based molecular subtypes, the authors demonstrated that patients with a high ICD score exhibited poorer survival, distinct immune profiles, and reduced responses to immunotherapy. Among the differentially expressed genes, HSP90AA1 emerged as uniquely upregulated in both high-ICD score tumors and in bladder cancer tissues compared to normal controls. High expression of HSP90AA1 was significantly associated with reduced disease-free survival, suggesting a link between its expression and aggressive tumor behavior. Intriguingly, HSP90AA1 was enriched in CD4^+^ T-cell subsets and positively correlated with immune checkpoint molecules such as PD-L1, indicating a role in shaping the immunosuppressive tumor microenvironment. The study confirmed that HSP90AA1, as a DAMP released during ICD, contributes to the activation of immune responses through interaction with pattern recognition receptors, but paradoxically, also supports tumor immune evasion in certain settings. Furthermore, pharmacogenomic screening identified several potential small-molecule inhibitors—alvespimycin, tanespimycin, and retaspimycin—that selectively target HSP90AA1 and may hold promise in sensitizing bladder cancer to immunotherapy. The dualistic role of HSP90AA1 emphasizes the need for targeted therapeutic strategies that can disrupt HSP90-mediated oncogenic signaling while harnessing its immunogenic potential to enhance antitumor immunity [108].

A recent study examining a pan-cancer perspective identifies HSP90B1 as a significant molecular chaperone involved in the progression of various malignancies, including bladder urothelial carcinoma. The authors demonstrated that HSP90B1 expression is significantly elevated in bladder cancer tissues compared to normal counterparts at both mRNA and protein levels. Importantly, high HSP90B1 expression in bladder urothelial carcinoma correlates with poor overall survival and disease-free survival, indicating its potential as a prognostic biomarker. Immunohistochemical data confirm cytoplasmic overexpression of HSP90B1 in bladder tumors, reinforcing its functional involvement in tumor pathophysiology. From an oncological perspective, HSP90B1 supports cancer progression by stabilizing oncogenic client proteins and participating in cellular stress responses, particularly endoplasmic reticulum homeostasis—functions that align with the canonical role of DAMPs in sustaining tumor cell survival under hostile microenvironmental conditions. Functional enrichment analysis of HSP90B1-interacting proteins revealed its integration into pathways regulating apoptosis, immune modulation, and ER stress, further suggesting its dual role in promoting oncogenesis and modulating anti-tumor immune responses. The involvement of HSP90B1 in immune cell infiltration, particularly its association with cancer-associated fibroblasts, underscores its broader influence on the tumor microenvironment. Taken together, these findings suggest that HSP90B1 may act as a pro-tumoral signaling and immune evasion molecule in bladder cancer. As such, it represents a promising therapeutic target and biomarker, warranting further investigation in the context of bladder cancer biology and treatment resistance. Two of the previously mentioned analyses show HSP90 family members associate with specific immune/inflammatory tumor microenvironments and with particular molecular subtypes. Extracellular HSP90 can reflect general cellular stress or immunogenic cell death rather than tumor-specific secretion, so comorbid inflammatory states could complicate interpretation. Reporting tumor-subtype specific performance is therefore important [108,109].

### 4.11. HMGB1 in Bladder Cancer

HMGB1 is another DAMP considered in the evaluation of its role in bladder tumors. Singh et al. analyzed tumor tissues and patient serum, demonstrating that HMGB1 is significantly upregulated at both the mRNA and protein levels in cancerous tissues compared to adjacent normal urothelium, implicating it in tumorigenic processes. Immunohistochemical staining revealed concurrent overexpression of HMGB1, RAGE, Beclin-1, and LC3, indicating an activated autophagic pathway within tumor cells, while p62 expression was downregulated, reflecting autophagic flux. These molecular alterations suggest that HMGB1 not only marks cellular stress and damage but may actively participate in autophagy-mediated tumor survival and progression. Importantly, serum HMGB1 levels were markedly higher in UBC patients and showed a positive correlation with tumor stage and tissue expression, underscoring its potential as a circulating DAMP capable of reflecting the tumor’s inflammatory and metabolic state. However, the study was small and cross-sectional (30 patients, 30 controls, limited tissue sample numbers for some assays), which constrains generalizability and prevents assessment of diagnostic performance over time or after treatment. Further large-scale and longitudinal studies are warranted to validate these findings and explore HMGB1 as a biomarker-integrated target in bladder cancer management [110].

Benlier et al. conducted an accurate study where they underscored the critical role of HMGB1 in bladder cancer. Significantly elevated urinary HMGB1 levels were observed in patients with bladder cancer compared to both healthy controls and those with urinary tract infections, indicating HMGB1’s potential as a discriminative marker. Importantly, HMGB1 concentrations correlated positively with tumor aggressiveness—patients with high-grade tumors, muscle-invasive disease, multifocal lesions, and larger tumor diameters (≥3 cm) exhibited markedly higher urinary HMGB1 levels. These findings suggest that HMGB1 secretion reflects not only tumor presence but also its biological behavior and invasiveness. From a diagnostic perspective, the study reported a urinary HMGB1 cut-off value of 63.3 pg/mL to differentiate bladder cancer from urinary tract infections, with a specificity of 77% and sensitivity of 59%, and an AUC of 0.813 against healthy controls—supporting its clinical potential. Notably, the highest HMGB1 levels were detected in patients presenting with concurrent high-grade, muscle-invasive, large, and multifocal tumors, highlighting its utility in risk stratification. Nevertheless, the sample was modest and from a single institution; sensitivity and specificity estimates were not validated in an independent cohort. The authors themselves note the need for larger, controlled, multicenter validation before clinical application. Given these multifaceted roles, HMGB1 emerges as a biomarker and a therapeutic target in bladder cancer. Benlier et al. compared urinary HMGB1 in bladder cancer vs. UTI very explicitly and found overlapping values, highlighting that infections and other inflammatory uropathies can raise HMGB1, and thus, reduce specificity; Singh et al. further showed tissue/serum correlations but emphasized the need for controls with benign inflammatory conditions. Therefore, future studies should include UTI/inflammatory control arms and adjust for markers of systemic inflammation [110,111].

The pan-cancer analysis conducted by Guan et al. showed the central role of HMGB1 in the progression and prognosis of various malignancies. In the context of bladder cancer, elevated HMGB1 expression also correlates with advanced tumor stages and poor patient outcomes. The study emphasized that HMGB1 not only serves as a biomarker for disease progression but also actively participates in modulating the tumor microenvironment. Furthermore, inhibiting HMGB1 function could disrupt its role in tumor-promoting pathways, thereby enhancing the efficacy of existing treatments and improving patient survival rates. The comprehensive insights provided by this study advocate for further investigation into HMGB1-targeted therapies in bladder cancer management [112].

### 4.12. S100A9 in Bladder Cancer

The final DAMP of interest is S100A9, which has been the subject of various studies. The study by Wang et al. highlighted the significant role of S100A9 in the progression and prognosis of bladder cancer. Through comprehensive analyses, including single-cell sequencing and functional assays, the research delineated the overexpression of S100A9 in MIBC compared to NMIBC. This overexpression correlates with enhanced tumor cell proliferation, migration, and invasion, suggesting that S100A9 contributes significantly to the aggressive phenotype observed in MIBC. The study’s findings also indicate that S100A9 may serve as a valuable prognostic biomarker, with its expression levels potentially guiding clinical decisions regarding patient management and therapeutic approaches. Further experiments demonstrated that reducing S100A9 expression impairs tumor cell proliferation and invasiveness, pointing to its viability as a therapeutic target [113].

**Figure 4:**
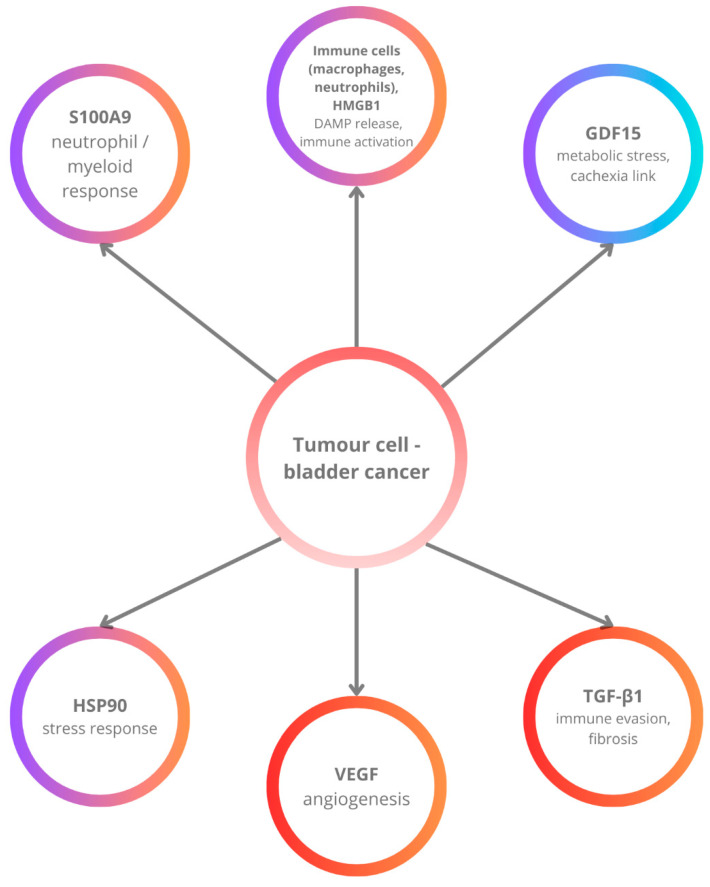
Simplified schematic overview of six emerging biomarkers in the bladder cancer microenvironment. Tumor cells engage angiogenic mediators (VEGF), immunomodulatory cytokines (TGF-*β*1), stress-response molecules (HSP90), and damage-associated molecular patterns (HMGB1, S100A9), while metabolic regulators such as GDF15 link tumor biology with systemic cachexia. Together, these factors contribute to angiogenesis, immune evasion, stromal remodeling, and inflammatory cell recruitment, facilitating progression.

Verma et al. conducted a study that identified S100A9 as a significant gene linked to the progression and prognosis of bladder cancer. Through integrated bioinformatics analyses of multiple gene expression datasets, S100A9 emerged among seven key genes differentially expressed between non-muscle-invasive and muscle-invasive bladder cancer specimens. Higher expression levels of S100A9 were observed in muscle-invasive bladder cancer tissues compared to non-muscle-invasive forms, indicating its role in tumor advancement. Consequently, tumor invasiveness and subtype significantly influence the levels of this biomarker, which should be considered when evaluating diagnostic specificity. Further validation using quantitative real-time PCR in various bladder cancer cell lines confirmed the elevated expression of S100A9, particularly in those representing advanced disease stages. Survival analyses indicated that increased S100A9 expression correlates with poorer overall survival, underscoring its potential as a prognostic biomarker. These findings highlight S100A9s involvement in bladder cancer pathogenesis and its promise as both a diagnostic and prognostic marker [114] [Figure 4].

Although each marker has some diagnostic or prognostic value individually, the reviewed evidence suggests that combinations are more promising. For bladder cancer, urinary HMGB1 plus VEGF appears particularly attractive, since they capture complementary processes and both are measurable in urine. Adding TGF-*β*1 could further enhance recurrence/progression prediction in NMIBC. For RCC, studies suggest that combining VEGF with S100A9 or HSP90 may yield better discrimination of aggressive disease. Panels that integrate angiogenesis-related factors (VEGF, TGF-*β*1) with DAMPs (HMGB1, S100A9) are therefore most promising, but require validation in prospective multicenter cohorts [Table 1].

### 4.13. Barriers to Clinical Implementation

Translation of biomarkers into routine clinical practice faces several non-biological but critical barriers. First, assay standardization and pre-analytical variability are major obstacles: different platforms (ELISA vs. multiplex immunoassay vs. mass spectrometry), choice of capture/detection antibodies, sample type (serum, plasma, urine), processing time, and storage conditions can produce systematic differences in measured concentrations and limit comparability between studies. Second, the reproducibility across laboratories is often unproven; few studies report inter-laboratory coefficients of variation or use reference materials that would enable unification. Third, threshold variability and population effects (age, co-morbidity, renal function, inflammation) mean that cut-offs derived in a single center may not generalize to other patient groups. Fourth, cost affects clinical uptake: an assay that is expensive requires specialized equipment is less likely to be adopted for screening or routine surveillance. Ultimately, regulatory and clinical-utility demonstration demands prospective, multicenter studies showing not only association with disease but other clinical benefits (e.g., improved diagnosis, decision making, or patient outcomes). For our six markers, these barriers translate to specific requirements: assay unification and reporting of analytical performance, pre-specified cut-off derivation in large, diverse cohorts, head-to-head comparisons using the same sample sets, and economic analyses of implementation.

In addition to analytical and clinical barriers, future implementation of biomarkers must be evaluated in terms of cost-effectiveness in real-world screening and surveillance programs. For bladder cancer, the greatest potential lies in reducing the need for repeated cystoscopies in low- and intermediate-risk patients. Even modest sensitivity improvements, if coupled with lower cost and non-invasiveness, could make urine-based biomarker panels economically favorable at the population level. For renal cancer, where imaging remains the standard, biomarkers could be tested as triage tools to reduce unnecessary imaging in high-risk cohorts. Formal health-economic models are, therefore, required to assess the balance between assay cost, diagnostic performance, and downstream healthcare savings.

Another factor to consider is the impact of systemic treatments on biomarker dynamics. VEGF and TGF-*β*1 are directly modulated by anti-angiogenic therapies and TGF-*β* pathway inhibitors, potentially confounding their diagnostic or prognostic interpretation in treated patients while simultaneously creating opportunities for pharmacodynamic monitoring. HMGB1 and S100A9, as immune-related DAMPs, may increase transiently after immune checkpoint blockade or chemotherapy due to treatment-induced tumor cell death and immune activation. HSP90, itself a therapeutic target, can decline under HSP90 inhibitor therapy. GDF15 is responsive to metabolic stress and may reflect treatment toxicity as much as tumor biology. These therapy-associated changes highlight the need to interpret biomarker levels in clinical context and to study on-treatment kinetics in prospective trials.

## 5. Conclusions

This review synthesizes current experimental and clinical evidence indicating that pro-angiogenic mediators and damage-associated molecular patterns represent complementary domains in biomarker research for urological malignancies. VEGF and TGF-*β*1 capture essential aspects of tumor angiogenesis, hypoxia signaling, and stromal remodeling, whereas danger signals such as HSP90, HMGB1 and S100A9 report on cellular stress, necrosis, and innate immune activation that shape the tumor microenvironment. GDF15 plays a unique role in connecting metabolic stress and susceptibility to ferroptosis with tumor behavior, especially in RCC. Together, these molecules illuminate multiple, intersecting biological processes that are relevant to diagnosis, prognostication, and therapy selection.

The accumulated data indicate several practical insights. Firstly, fluid-based measurements—notably urinary HMGB1 and urinary VEGF in bladder cancer and serum or tissue assessments of GDF15, HSP90, and S100A9 in renal tumors—provide viable, minimally invasive opportunities to complement imaging and histopathology. Secondly, single analyses provide useful biological insight but often lack adequate accuracy alone; combined assessments of angiogenic, immunomodulatory, and damage-associated markers yield greater discriminatory and prognostic value. Thirdly, the biological roles of these factors are context-dependent: TGF-*β*1 can exert tumor-suppressive effects early in carcinogenesis yet promote invasion and EMT in advanced disease, while DAMPs may both alert the immune system and drive immunosuppressive inflammation depending on local conditions.

Despite promising signals, important barriers remain before routine clinical adoption. Heterogeneity across tumor subtypes and patient comorbidities, variability in assay platforms and pre-analytical handling, and the predominance of retrospective, single-center series limit generalization. Critical needs include harmonization of laboratory methods, definition of clinically meaningful thresholds, validation of marker performance in prospectively collected, multicenter cohorts, and careful evaluation of intra-individual longitudinal variability during treatment and follow-up.

Future work should pursue two mutually reinforcing paths. Future studies must clarify causal links—for example, how modulation of GDF15 influences ferroptosis in ccRCC, or how extracellular HSP90 and HMGB1 alter antigen presentation and local immune composition. Simultaneously, well-designed prospective clinical studies and interventional trials should test whether integrated biomarker assessment can improve detection of early or recurrent disease, refine risk stratification between indolent and aggressive phenotypes, and guide therapeutic choices (including strategies that target angiogenesis, immune suppression, or stress-response pathways). Importantly, the development of validated, clinically applicable multi-marker panels combined with standard imaging offers the most promising route to translate these molecular insights into patient benefit.

In summary, angiogenic mediators and DAMP-related factors provide complementary, biologically grounded information about urological tumors. They hold substantial promise as adjuncts to current diagnostic and monitoring pathways, but their clinical value will depend on rigorous standardization and prospective validation. Only through such coordinated translational efforts these biomarkers can move from exploratory studies to reliable tools that improve outcomes for patients with renal and bladder cancer.

## Figures and Tables

**Table 1:** Summary of proposed biomarkers for urological cancers (based on [94,95,96,97,98,99,100,103,104,105,106,107,108,109,110,111,112,113,114]).

Cancer Type	Proposed Biomarker/Panel	Potential Clinical Application & Key Findings
RCC	GDF15 + HSP90	Prognosis and therapeutic stratification: correlation with ferroptosis susceptibility in RCC, suggesting a potential role in predicting response to therapies that modulate this cell death pathway.
	urinary HMGB1	Diagnosis and prognosis: functions as a non-invasive biomarker. Levels are markedly elevated in RCC patients and correlate strongly with tumor size and high-grade, aggressive disease, offering both diagnostic and prognostic value.
	S100A9	Early detection and prognosis: overexpressed in the serum and tissues of early-stage RCC, indicating potential as an early diagnostic marker. High tissue expression is an independent predictor of poor disease-free and disease-specific survival.
	integrated panel of VEGF, TGF-*β*1, DAMPs	Early detection and therapy monitoring: a proposed combination of pro-angiogenic (VEGF), immunomodulatory (TGF-*β*1), and damage-associated (e.g., HMGB1, S100A9) markers with advanced imaging could refine early detection and enable dynamic monitoring of therapeutic response.
bladder cancer	urinary VEGF + urinary HMGB1	Non-invasive detection: the combination of these urinary biomarkers is suggested to enhance the non-invasive detection of bladder cancer. Urinary VEGF is significantly elevated in patients, and urinary HMGB1 levels correlate with tumor aggressiveness.
	serum TGF-*β*1 + serum VEGF	Staging and diagnosis: The combined measurement of serum TGF-*β*1 and VEGF showed superior diagnostic performance in distinguishing MIBC from non-muscle-invasive disease compared to either marker alone.
	S100A9	Prognosis and progression prediction: increased S100A9 expression is associated with muscle-invasive disease and poorer overall survival.

Abbreviations: RCC (renal cell carcinoma), GDF15 (growth differentiation factor 15), HSP90 (heat shock protein 90), HMGB1 (high mobility group box 1), S100A9 (S100 calcium-binding protein A9), VEGF (vascular endothelial growth factor), TGF-*β*1 (transforming growth factor beta 1), DAMPs (damage-associated molecular patterns), MIBC (muscle-invasive bladder cancer). All biomarkers were evaluated for their diagnostic, prognostic, or therapeutic potential in urological cancers based on recent studies.

## Data Availability

No new data were created or analyzed in this study.
